# Crystal structure of a third polymorph of tris­(acetyl­acetonato-κ^2^ *O*,*O*′)iron(III)

**DOI:** 10.1107/S2056989015021805

**Published:** 2015-11-21

**Authors:** Tessa M. Baker, Kevin M. Howard, William W. Brennessel, Michael L. Neidig

**Affiliations:** a Department of Chemistry, 120 Trustee Road, University of Rochester, Rochester, NY 14627, USA

**Keywords:** crystal structure, twin, polymorphism, ferric acetyl­acetonate

## Abstract

In the structure of the title complex, [Fe(C_5_H_7_O_2_)_3_] or Fe(acac)_3_, the asymmetric unit contains one mol­ecule in a general position. The coordination sphere of the Fe^III^ atom is that of a slightly distorted octahedron. The crystal under investigation was a two-component pseudo-merohedral twin in the monoclinic system with a β angle close to 90°. Twin law [100/0-10/00-1] reduced the *R*1 residual [*I* > 2*σ*(*I*)] from 0.0769 to 0.0312, and the mass ratio of twin components refined to 0.8913 (5):0.1087 (5). In the crystal, mol­ecules are arranged in sheets normal to [001] *via* non-classical C—H⋯O hydrogen bonding. No other significant inter­molecular inter­actions are observed. The structure is a new polymorph of Fe(acac)_3_ and is isotypic with one polymorph of its gallium analog.

## Related literature   

For an early report of the first polymorph of tris­(acetyl­acetonato)iron(III), see: Morgan & Drew (1921 ▸), and references therein. For a later occurrence of this polymorph, see: Molokhia *et al.* (1981 ▸). For multiple reports of the second polymorph, see: Roof (1956 ▸); Shkol’nikova (1959 ▸); Iball & Morgan (1967 ▸); Kabak *et al.* (1996 ▸); Diaz-Acosta *et al.* (2001 ▸); Hu *et al.* (2001 ▸); Stabnikov *et al.* (2007 ▸); Weng *et al.* (2011 ▸). For the isotypic gallium analog, see: Sultan *et al.* (2005 ▸).
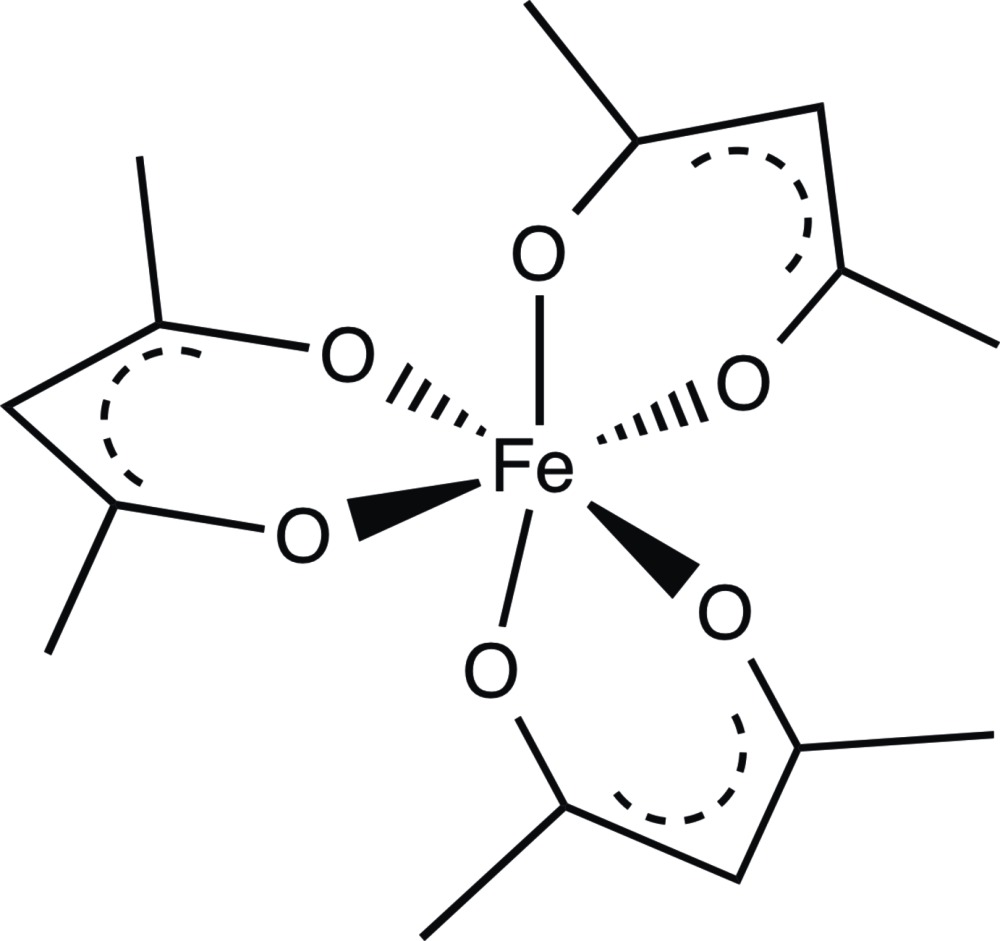


## Experimental   

### Crystal data  

[Fe(C_5_H_7_O_2_)_3_]
*M* *_r_* = 353.17
Monoclinic, 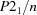

*a* = 8.011 (3) Å
*b* = 13.092 (5) Å
*c* = 15.808 (6) Å
β = 90.108 (7)°
*V* = 1658.1 (10) Å^3^
*Z* = 4
Mo *K*α radiation
μ = 0.93 mm^−1^
*T* = 100 K
0.48 × 0.20 × 0.06 mm

### Data collection  

Bruker SMART APEXII CCD platform diffractometer
Absorption correction: multi-scan (*SADABS*; Sheldrick, 2014 ▸) *T*_min_ = 0.642, *T*_max_ = 0.748
52218 measured reflections
9058 independent reflections
7693 reflections with *I* > 2σ(*I*)
*R*_int_ = 0.041

### Refinement  

*R*[*F* ^2^ > 2σ(*F* ^2^)] = 0.031
*wR*(*F* ^2^) = 0.081
*S* = 1.06
9058 reflections
206 parameters
H-atom parameters constrained
Δρ_max_ = 0.50 e Å^−3^
Δρ_min_ = −0.58 e Å^−3^

### 

Data collection: *APEX2* (Bruker, 2014 ▸); cell refinement: *SAINT* (Bruker, 2013 ▸); data reduction: *SAINT*; program(s) used to solve structure: *SHELXS2013* (Sheldrick, 2008 ▸); program(s) used to refine structure: *SHELXL2014* (Sheldrick, 2015 ▸); molecular graphics: *SHELXTL* (Sheldrick, 2008 ▸); software used to prepare material for publication: *SHELXTL*.

## Supplementary Material

Crystal structure: contains datablock(s) I, global. DOI: 10.1107/S2056989015021805/vn2103sup1.cif

Structure factors: contains datablock(s) I. DOI: 10.1107/S2056989015021805/vn2103Isup2.hkl

**Fig. 1.**
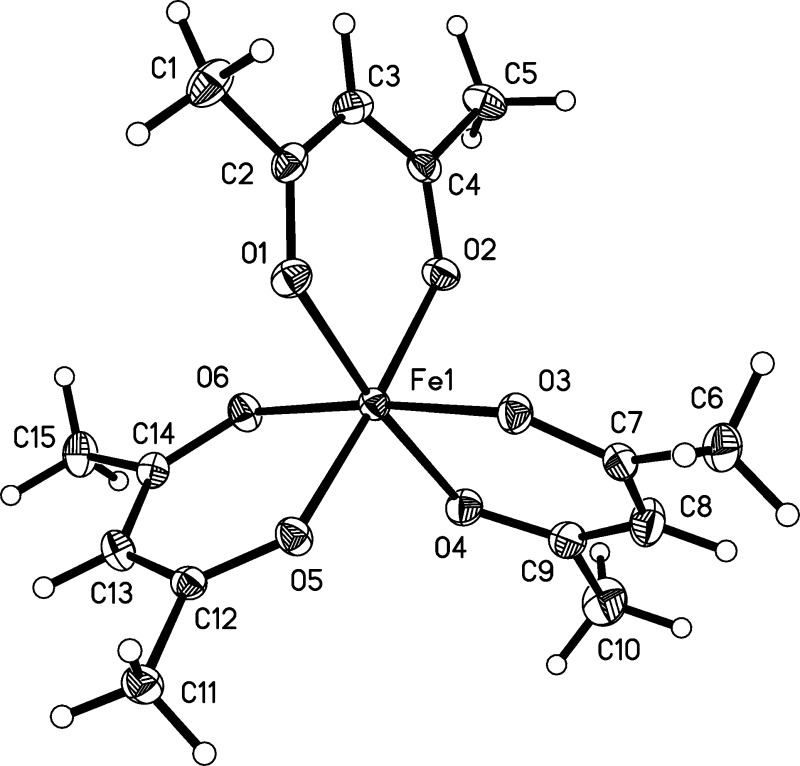
The structure of the molecule showing the atom numbering, with displacement ellipsoids drawn at the 50% probability level.

. DOI: 10.1107/S2056989015021805/vn2103fig1.tif
The structure of the mol­ecule showing the atom numbering, with displacement ellipsoids drawn at the 50% probability level.

CCDC reference: 1437249

Additional supporting information:  crystallographic information; 3D view; checkCIF report

## Figures and Tables

**Table 1:** Selected bond lengths (Å)

Fe1—O5	1.9874 (9)
Fe1—O2	1.9986 (9)
Fe1—O4	1.9987 (9)
Fe1—O6	2.0008 (9)
Fe1—O1	2.0063 (9)
Fe1—O3	2.0098 (10)

**Table 2:** Hydrogen-bond geometry (Å, °)

*D*—H⋯*A*	*D*—H	H⋯*A*	*D*⋯*A*	*D*—H⋯*A*
C11—H11*C*⋯O3^i^	0.98	2.60	3.4736 (15)	148
C15—H15*C*⋯O3^ii^	0.98	2.47	3.4326 (15)	167

Symmetry codes: (i) 
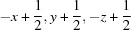
; (ii) 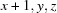.

## supplementary crystallographic information

### S1. Comment

To date crystal structures of the unsolvated title complex (Figure 1) have only appeared in one of two polymorphic forms, and both are orthorhombic. The original report of the first polymorph was described by von Lang in 1899 (Morgan & Drew, 1921, and references therein), and was reported again over 80 years later (Molokhia *et al.*, 1981). The second polymorph has been presented in multiple publications (Roof Jr, 1956, Shkol'nikova, 1959, Iball & Morgan, 1967, Kabak *et al.*, 1996, Diaz-Acosta *et al.*, 2001, Hu *et al.*, 2001, Stabnikov *et al.*, 2007, and Weng *et al.*, 2011). This report presents a new (third) polymorph for the iron complex. The structure is isotypic with one polymorph of its gallium analog (Sultan *et al.*, 2005).

Although the beta angle of the title compound is very close to 90°, the data are truly monoclinic. Because of the near-90° beta angle, the potential for twinning existed, and indeed, the crystal was a pseudo-merohedral twin. Upon completion of the experiment at 100 K, additional sets of data were collected at room temperature to check for any phase changes, of which there were none. Attempts to reproduce the crystallization of this polymorph have been unsuccessful to date.

### S2. Experimental

Large flat red rectangular prisms grew over the course of weeks from the slow evaporation of a diethyl ether solution at 243 K.

### S3. Refinement

H atoms were placed geometrically and treated as riding atoms: C—H(*sp*^2^) = 0.95 Å with *U*_iso(_H) = 1.2*U*_eq_(C) and *C*—*H*(methyl) = 0.98 Å with *U*_iso(_H) = 1.5*U*_eq_(C).

### Crystal data

**Table:**

[Fe(C_5_H_7_O_2_)_3_]	*F*(000) = 740
*M**_r_* = 353.17	*D*_x_ = 1.415 Mg m^−^^3^
Monoclinic, *P*2_1_/*n*	Mo *K*α radiation, λ = 0.71073 Å
*a* = 8.011 (3) Å	Cell parameters from 3703 reflections
*b* = 13.092 (5) Å	θ = 2.9–37.9°
*c* = 15.808 (6) Å	µ = 0.93 mm^−^^1^
β = 90.108 (7)°	*T* = 100 K
*V* = 1658.1 (10) Å^3^	Rectangular prism, red
*Z* = 4	0.48 × 0.20 × 0.06 mm

### Data collection

**Table:**

Bruker SMART APEXII CCD platform diffractometer	7693 reflections with *I* > 2σ(*I*)
Radiation source: fine-focus sealed tube	*R*_int_ = 0.041
ω scans	θ_max_ = 38.6°, θ_min_ = 1.3°
Absorption correction: multi-scan (*SADABS*; Sheldrick, 2014)	*h* = −14→13
*T*_min_ = 0.642, *T*_max_ = 0.748	*k* = −22→22
52218 measured reflections	*l* = −27→27
9058 independent reflections	

### Refinement

**Table:**

Refinement on *F*^2^	Primary atom site location: heavy-atom method
Least-squares matrix: full	Secondary atom site location: difference Fourier map
*R*[*F*^2^ > 2σ(*F*^2^)] = 0.031	Hydrogen site location: inferred from neighbouring sites
*wR*(*F*^2^) = 0.081	H-atom parameters constrained
*S* = 1.06	*w* = 1/[σ^2^(*F*_o_^2^) + (0.0422*P*)^2^ + 0.1283*P*] where *P* = (*F*_o_^2^ + 2*F*_c_^2^)/3
9058 reflections	(Δ/σ)_max_ = 0.002
206 parameters	Δρ_max_ = 0.50 e Å^−^^3^
0 restraints	Δρ_min_ = −0.58 e Å^−^^3^

### Special details

**Table:**

Geometry. All e.s.d.'s (except the e.s.d. in the dihedral angle between two l.s. planes) are estimated using the full covariance matrix. The cell e.s.d.'s are taken into account individually in the estimation of e.s.d.'s in distances, angles and torsion angles; correlations between e.s.d.'s in cell parameters are only used when they are defined by crystal symmetry. An approximate (isotropic) treatment of cell e.s.d.'s is used for estimating e.s.d.'s involving l.s. planes.
Refinement. The crystal was a two-component pseudo-merohedral twin. Twin law [1 0 0 / 0 - 1 0 / 0 0 - 1] reduced the R1 residual (observed) from 0.0769 to 0.0312. The mass ratio of twin components refined to 0.8913 (5):0.1087 (5).

### Fractional atomic coordinates and isotropic or equivalent isotropic displacement parameters (Å^2^)

**Table:**

	*x*	*y*	*z*	*U*_iso_*/*U*_eq_	
Fe1	0.45028 (2)	0.51493 (2)	0.25157 (2)	0.01370 (3)	
O1	0.34731 (10)	0.56636 (5)	0.35904 (4)	0.01882 (13)	
O2	0.45230 (9)	0.37683 (5)	0.30543 (4)	0.01803 (12)	
O3	0.22215 (9)	0.49718 (5)	0.20055 (5)	0.01809 (12)	
O4	0.54074 (9)	0.44882 (6)	0.14732 (4)	0.01907 (12)	
O5	0.44928 (9)	0.65608 (5)	0.20532 (4)	0.01776 (12)	
O6	0.68337 (9)	0.54067 (5)	0.29194 (5)	0.01786 (12)	
C1	0.24199 (14)	0.58184 (9)	0.49814 (6)	0.02391 (19)	
H1A	0.2763	0.6533	0.4922	0.036*	
H1B	0.2799	0.5555	0.5529	0.036*	
H1C	0.1201	0.5773	0.4948	0.036*	
C2	0.31834 (12)	0.51942 (7)	0.42813 (6)	0.01747 (15)	
C3	0.34901 (13)	0.41562 (8)	0.44180 (6)	0.01941 (16)	
H3A	0.3256	0.3883	0.4962	0.023*	
C4	0.41212 (11)	0.34971 (7)	0.38019 (6)	0.01657 (15)	
C5	0.43362 (15)	0.23768 (8)	0.39930 (7)	0.02404 (19)	
H5A	0.5452	0.2157	0.3818	0.036*	
H5B	0.3492	0.1983	0.3684	0.036*	
H5C	0.4204	0.2262	0.4602	0.036*	
C6	−0.00658 (14)	0.44499 (9)	0.11612 (7)	0.0257 (2)	
H6A	−0.0578	0.5103	0.1317	0.039*	
H6B	−0.0571	0.3899	0.1494	0.039*	
H6C	−0.0249	0.4320	0.0558	0.039*	
C7	0.17850 (12)	0.44918 (7)	0.13395 (6)	0.01805 (16)	
C8	0.28949 (14)	0.40143 (9)	0.07851 (7)	0.0253 (2)	
H8A	0.2443	0.3651	0.0318	0.030*	
C9	0.46286 (14)	0.40398 (7)	0.08771 (6)	0.02005 (16)	
C10	0.57164 (17)	0.35230 (10)	0.02260 (8)	0.0309 (2)	
H10A	0.6496	0.4022	−0.0013	0.046*	
H10B	0.5014	0.3243	−0.0226	0.046*	
H10C	0.6346	0.2969	0.0495	0.046*	
C11	0.51133 (13)	0.82920 (7)	0.17750 (6)	0.01980 (17)	
H11A	0.4886	0.8213	0.1169	0.030*	
H11B	0.6042	0.8770	0.1855	0.030*	
H11C	0.4116	0.8558	0.2058	0.030*	
C12	0.55669 (12)	0.72745 (6)	0.21471 (5)	0.01511 (14)	
C13	0.71029 (11)	0.71624 (7)	0.25599 (6)	0.01816 (15)	
H13A	0.7801	0.7746	0.2609	0.022*	
C14	0.76718 (11)	0.62403 (7)	0.29054 (6)	0.01557 (14)	
C15	0.93944 (13)	0.61963 (8)	0.32810 (7)	0.02235 (18)	
H15A	0.9331	0.5932	0.3860	0.034*	
H15B	0.9879	0.6884	0.3289	0.034*	
H15C	1.0097	0.5744	0.2939	0.034*	

### Atomic displacement parameters (Å^2^)

**Table:**

	*U*^11^	*U*^22^	*U*^33^	*U*^12^	*U*^13^	*U*^23^
Fe1	0.01298 (5)	0.01363 (5)	0.01448 (5)	−0.00062 (4)	0.00053 (5)	−0.00014 (4)
O1	0.0206 (3)	0.0188 (3)	0.0170 (3)	0.0029 (2)	0.0013 (2)	−0.0015 (2)
O2	0.0210 (3)	0.0148 (3)	0.0183 (3)	−0.0001 (2)	0.0040 (2)	0.0009 (2)
O3	0.0150 (3)	0.0211 (3)	0.0182 (3)	−0.0018 (2)	−0.0002 (2)	−0.0028 (2)
O4	0.0180 (3)	0.0215 (3)	0.0177 (3)	0.0000 (2)	0.0024 (2)	−0.0022 (2)
O5	0.0155 (3)	0.0167 (3)	0.0210 (3)	−0.0015 (2)	−0.0029 (2)	0.0028 (2)
O6	0.0151 (3)	0.0159 (3)	0.0225 (3)	−0.0001 (2)	−0.0033 (2)	0.0012 (2)
C1	0.0207 (4)	0.0325 (5)	0.0185 (4)	0.0061 (4)	0.0001 (3)	−0.0066 (4)
C2	0.0132 (3)	0.0235 (4)	0.0157 (3)	0.0009 (3)	−0.0012 (3)	−0.0034 (3)
C3	0.0198 (4)	0.0231 (4)	0.0154 (3)	0.0001 (3)	0.0015 (3)	0.0013 (3)
C4	0.0137 (3)	0.0176 (3)	0.0184 (4)	−0.0023 (3)	−0.0003 (3)	0.0023 (3)
C5	0.0267 (5)	0.0183 (4)	0.0272 (5)	−0.0005 (3)	0.0032 (4)	0.0065 (3)
C6	0.0187 (4)	0.0345 (5)	0.0238 (5)	−0.0066 (4)	−0.0048 (4)	−0.0005 (4)
C7	0.0186 (4)	0.0165 (3)	0.0190 (4)	−0.0037 (3)	−0.0020 (3)	0.0015 (3)
C8	0.0241 (5)	0.0283 (5)	0.0235 (4)	−0.0038 (4)	−0.0006 (4)	−0.0093 (4)
C9	0.0250 (5)	0.0168 (4)	0.0184 (4)	0.0000 (3)	0.0038 (3)	−0.0028 (3)
C10	0.0331 (6)	0.0315 (5)	0.0281 (5)	0.0022 (5)	0.0081 (4)	−0.0113 (4)
C11	0.0236 (4)	0.0156 (3)	0.0202 (4)	0.0012 (3)	0.0002 (3)	0.0019 (3)
C12	0.0167 (4)	0.0142 (3)	0.0144 (3)	0.0006 (3)	0.0031 (3)	−0.0004 (2)
C13	0.0150 (3)	0.0153 (3)	0.0241 (4)	−0.0014 (3)	−0.0002 (3)	−0.0008 (3)
C14	0.0129 (3)	0.0173 (3)	0.0165 (3)	0.0010 (3)	0.0005 (3)	−0.0027 (3)
C15	0.0147 (4)	0.0240 (4)	0.0283 (4)	0.0009 (3)	−0.0047 (3)	−0.0041 (3)

### Geometric parameters (Å, º)

**Table:**

Fe1—O5	1.9874 (9)	C6—C7	1.5097 (16)
Fe1—O2	1.9986 (9)	C6—H6A	0.9800
Fe1—O4	1.9987 (9)	C6—H6B	0.9800
Fe1—O6	2.0008 (9)	C6—H6C	0.9800
Fe1—O1	2.0063 (9)	C7—C8	1.3973 (15)
Fe1—O3	2.0098 (10)	C8—C9	1.3967 (17)
O1—C2	1.2747 (13)	C8—H8A	0.9500
O2—C4	1.2759 (12)	C9—C10	1.5100 (15)
O3—C7	1.2745 (12)	C10—H10A	0.9800
O4—C9	1.2726 (12)	C10—H10B	0.9800
O5—C12	1.2788 (12)	C10—H10C	0.9800
O6—C14	1.2816 (12)	C11—C12	1.5006 (13)
C1—C2	1.5065 (14)	C11—H11A	0.9800
C1—H1A	0.9800	C11—H11B	0.9800
C1—H1B	0.9800	C11—H11C	0.9800
C1—H1C	0.9800	C12—C13	1.3994 (14)
C2—C3	1.3979 (15)	C13—C14	1.4010 (13)
C3—C4	1.3966 (14)	C13—H13A	0.9500
C3—H3A	0.9500	C14—C15	1.5024 (14)
C4—C5	1.5073 (14)	C15—H15A	0.9800
C5—H5A	0.9800	C15—H15B	0.9800
C5—H5B	0.9800	C15—H15C	0.9800
C5—H5C	0.9800		
			
O5—Fe1—O2	176.37 (3)	C7—C6—H6A	109.5
O5—Fe1—O4	95.77 (4)	C7—C6—H6B	109.5
O2—Fe1—O4	87.52 (4)	H6A—C6—H6B	109.5
O5—Fe1—O6	87.93 (3)	C7—C6—H6C	109.5
O2—Fe1—O6	90.56 (3)	H6A—C6—H6C	109.5
O4—Fe1—O6	89.79 (4)	H6B—C6—H6C	109.5
O5—Fe1—O1	89.89 (3)	O3—C7—C8	124.38 (10)
O2—Fe1—O1	86.90 (3)	O3—C7—C6	116.10 (9)
O4—Fe1—O1	173.63 (3)	C8—C7—C6	119.51 (9)
O6—Fe1—O1	93.35 (4)	C9—C8—C7	123.91 (9)
O5—Fe1—O3	87.53 (3)	C9—C8—H8A	118.0
O2—Fe1—O3	94.18 (3)	C7—C8—H8A	118.0
O4—Fe1—O3	87.13 (4)	O4—C9—C8	125.04 (9)
O6—Fe1—O3	174.22 (3)	O4—C9—C10	115.38 (10)
O1—Fe1—O3	90.21 (4)	C8—C9—C10	119.57 (10)
C2—O1—Fe1	129.75 (7)	C9—C10—H10A	109.5
C4—O2—Fe1	130.12 (6)	C9—C10—H10B	109.5
C7—O3—Fe1	129.53 (7)	H10A—C10—H10B	109.5
C9—O4—Fe1	129.22 (7)	C9—C10—H10C	109.5
C12—O5—Fe1	129.38 (6)	H10A—C10—H10C	109.5
C14—O6—Fe1	128.80 (6)	H10B—C10—H10C	109.5
C2—C1—H1A	109.5	C12—C11—H11A	109.5
C2—C1—H1B	109.5	C12—C11—H11B	109.5
H1A—C1—H1B	109.5	H11A—C11—H11B	109.5
C2—C1—H1C	109.5	C12—C11—H11C	109.5
H1A—C1—H1C	109.5	H11A—C11—H11C	109.5
H1B—C1—H1C	109.5	H11B—C11—H11C	109.5
O1—C2—C3	124.68 (9)	O5—C12—C13	124.65 (8)
O1—C2—C1	116.28 (9)	O5—C12—C11	116.15 (9)
C3—C2—C1	119.03 (9)	C13—C12—C11	119.20 (8)
C4—C3—C2	123.82 (9)	C12—C13—C14	123.87 (8)
C4—C3—H3A	118.1	C12—C13—H13A	118.1
C2—C3—H3A	118.1	C14—C13—H13A	118.1
O2—C4—C3	124.50 (9)	O6—C14—C13	124.79 (9)
O2—C4—C5	115.31 (9)	O6—C14—C15	116.19 (8)
C3—C4—C5	120.19 (9)	C13—C14—C15	119.01 (8)
C4—C5—H5A	109.5	C14—C15—H15A	109.5
C4—C5—H5B	109.5	C14—C15—H15B	109.5
H5A—C5—H5B	109.5	H15A—C15—H15B	109.5
C4—C5—H5C	109.5	C14—C15—H15C	109.5
H5A—C5—H5C	109.5	H15A—C15—H15C	109.5
H5B—C5—H5C	109.5	H15B—C15—H15C	109.5
			
Fe1—O1—C2—C3	−2.73 (15)	Fe1—O4—C9—C8	7.50 (16)
Fe1—O1—C2—C1	178.71 (7)	Fe1—O4—C9—C10	−173.67 (8)
O1—C2—C3—C4	−1.55 (16)	C7—C8—C9—O4	0.58 (19)
C1—C2—C3—C4	176.97 (10)	C7—C8—C9—C10	−178.20 (11)
Fe1—O2—C4—C3	2.61 (14)	Fe1—O5—C12—C13	5.79 (14)
Fe1—O2—C4—C5	−178.63 (7)	Fe1—O5—C12—C11	−174.69 (6)
C2—C3—C4—O2	1.63 (16)	O5—C12—C13—C14	1.60 (15)
C2—C3—C4—C5	−177.08 (9)	C11—C12—C13—C14	−177.90 (9)
Fe1—O3—C7—C8	−3.57 (15)	Fe1—O6—C14—C13	−2.54 (14)
Fe1—O3—C7—C6	176.19 (7)	Fe1—O6—C14—C15	178.36 (7)
O3—C7—C8—C9	−2.61 (18)	C12—C13—C14—O6	−3.25 (16)
C6—C7—C8—C9	177.64 (11)	C12—C13—C14—C15	175.82 (9)

### Hydrogen-bond geometry (Å, º)

**Table:**

*D*—H···*A*	*D*—H	H···*A*	*D*···*A*	*D*—H···*A*
C11—H11*C*···O3^i^	0.98	2.60	3.4736 (15)	148
C15—H15*C*···O3^ii^	0.98	2.47	3.4326 (15)	167

Symmetry codes: (i) −*x*+1/2, *y*+1/2, −*z*+1/2; (ii) *x*+1, *y*, *z*.
